# Papillary Thyroid Carcinoma with Nodular Fasciitis-Like Stroma in a 28-Year-Old Patient

**DOI:** 10.30699/IJP.2022.139405.2525

**Published:** 2022-03-08

**Authors:** Vahid Zand, Mansour Moghimi, Elmira Sadeghi, Pegah Kamal, Sedighe Vaziribozorg

**Affiliations:** 1 Department of Otolaryngology-Head and Neck Surgery, Otorhinolaryngology Research Center, Shahid Sadoughi University of Medical Sciences, Yazd, Iran; 2 Department of Pathology, Shahid Sadoughi University of Medical Sciences, Yazd, Iran

**Keywords:** Fibromatosis-like stroma, Neck mass, Papillary thyroid carcinoma

## Abstract

Papillary thyroid carcinoma (PTC) is considered as a relatively common type of malignancy showing a wide morphologic spectrum. Different variants of this tumor have been reported. Among PTC variants, PTC with nodular fasciitis-like stroma (PTCFLS) is rare. This variant consists of stromal components rich in spindle cells and accounts for 60-80% of tumors. In addition, there are small foci of epithelial components in PTCFLS though its features are similar to conventional PTC. In this case study, we present a new case with PTCFLS. The case is a 28-year-old female who was referred to the ENT clinic due to a painless mass on the anterior part of her neck. The mass showed a gradual increase in size over the 6 months prior to her referral. Thyroid test results were normal. Ultrasound imaging demonstrated an 84 × 36 mm heterogeneous nodule in the right thyroid lobe without calcifications but increased vascularity. There were also some reactive lymph nodes in both sub-mandibular areas. An ultrasound-guided fine-needle aspiration (FNA) biopsy of the right thyroid lobe nodule revealed a benign thyroid adenomatoid nodule. Following right thyroid lobectomy, final pathologic studies confirmed a diagnosis of PTC with exuberant fibromatosis-like stroma. In the 20-day post-surgery visit, the patient was found asymptomatic. Re-evaluation of the left thyroid lobe and follow-up were recommended. In this study, a diagnosis of a rare variant of PTC, i.e., PTC-FLS, was made through a combination of ultrasonography, fine needle aspiration cytology, and histological examination.

## Introduction

As a common carcinoma, papillary thyroid carcino-ma (PTC) has shown an increase in the incidence in several countries over the recent decades (1). This carcinoma has a wide morphologic spectrum, and different variants of this tumor have been reported (2). PTC with nodular fasciitis-like stroma (PTCFLS) is rare. Only 0.1%-0.5% of PTC cases develop to PTCFLS (2). Usually, there is a history of surgical trauma in 63% of cases of PTCFLS (3). PTCFLS is generally manifested in 20 to 82 years old (mean, 45 years), while it is more common in females (3:1). Mostly, the patients presented with a palpable neck mass (4). Histologically, this variant consists of stromal components rich in spindle cells and accounts for 60-80% of tumors. In addition, there are small foci of epithelial components in PTCFLS though its features are similar to conventional PTC. Findings of ultrastructural and immunohistochemical observations have revealed the presence of spindle cells in the tumor stroma with myofibroblast-like characteristics (4)**.** Complete surgical resection is the main therapy for PTCFLS, but this is difficult to achieve in the head and neck region while preserving complex and vital anatomy (5). Local excision and radiation therapy can be used for patients who are not willing to have surgery (6). In the present study, a female patient with PTCFLS is reported.

## Case Presentation

**Fig. 1:**
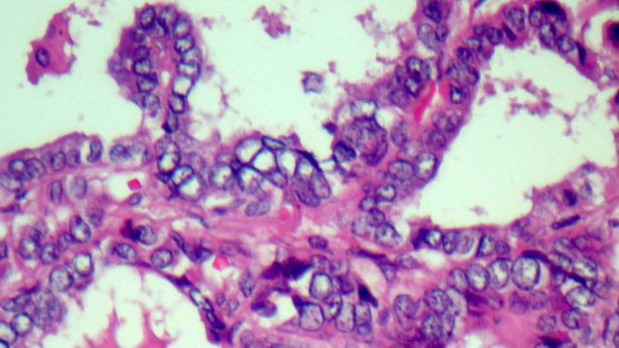
Hematoxylin & Eosin (HE) staining, magnification, 40×. Papillary architecture with overlapping, irregular, and ground-glass nuclei

**Fig. 2:**
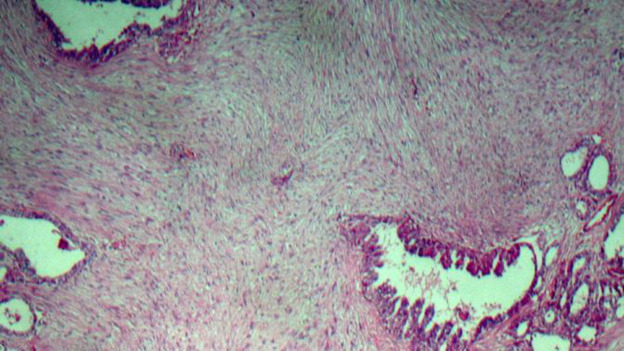
Hematoxylin & Eosin (HE) staining, magnification, 10×. Stromal and epithelial components. Spindle cells are arranged in reticulated fascicles with bland-looking nuclei

**Fig. 3:**
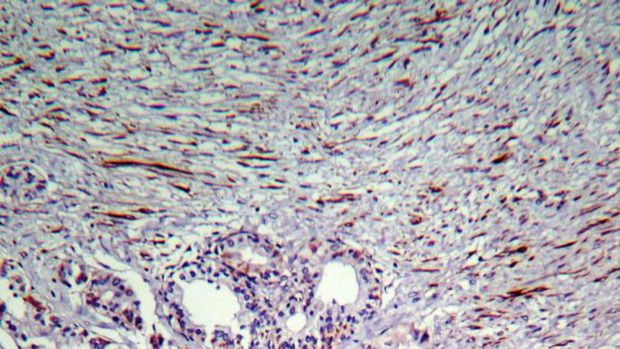
Immunohistochemical (IHC) staining, 10×. Spindle cells were diffusely and strongly positive for desmin

**Fig. 4:**
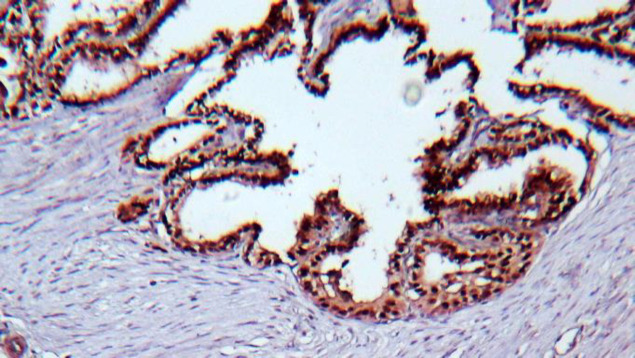
Immunohistochemical (IHC) staining, 40×.TTF1 was expressed diffusely in the epithelial component

A 28-year-old female was referred to the ENT clinic due to a painless mass on the anterior part of her neck. The mass had shown a gradual increase in size over 6 months. There was no positive family medical history. Thyroid test results were normal, but anemia was reported. Ultrasound showed an 84×36 mm heterogeneous nodule in the right thyroid lobe without calcifications with increased vascularity and some reactive lymph nodes in sub-mandibular areas (both sides) and right Jogular chain (Maximum size = 16×6 mm). An ultrasound-guided fine-needle aspiration (FNA) biopsy of the right thyroid lobe nodule revealed hypercellular smears composing of many monolayer sheets of follicular cells with mainly microfollicular structure and some papillary like structure with anisonuclei, some hemosiderin-laden macrophages distributed in the background of bloody and some amount of colloid material suggestive for a benign thyroid adenomatoid nodule (Figure 1). Right thyroid lobectomy was performed. The diagnosis was complicated since there were a few scattered tumor islands and extensive fibrosis in our provided slides. The specimen was firm without foci of calcification and adhesion. On gross examination, the measurement of the right thyroid lobe was 8 cm × 6 cm × 3 cm. Serial sectioning showed a gray-white cut surface with focal brownish areas, and the thyroid parenchyma with a diameter of 1.5 cm was seen at the margin of the sample. Histopathological examination of the right thyroid lobe showed a neoplastic lesion consisting of stromal and epithelial components. The epithelial component revealed features of conventional PTC, which consisted of a proliferation of small follicles and a few papilla structures lined by cuboidal and columnar cells and surrounded by a stromal component. These cells showed pale to ground-glass nuclei with some intranuclear inclusions, and grooves were also seen (Figure 2). Approximately 70% of the tumor mass was composed of a prominent proliferation of fibroblast cells exhibiting elongated, vesicular, and bland-looking nuclei with fine chromatin, a small distinct nucleolus, and indistinct cytoplasmic membrane that were arranged in reticulated fascicles by thick collagen fibers (Figure 3). Scattered chronic inflammatory cells were also seen in the tumor stroma. Immuno-histochemical (IHC) staining was performed for and demonstrated positivity of the stromal spindle cells with Desmin, while cuboidal-columnar cells of epithelial components were stained with TTF1 (Figure 4). Final pathologic studies confirmed a diagnosis of “Papillary thyroid carcinoma with exuberant fibromatosis-like stroma”. Twenty days after the examination, the patient was asymptomatic, and re-evaluation of the left thyroid lobe and follow-up were recommended.

## Discussion

Recently, PTC-FLS has been identified as a rare variant of PTC. To make such a diagnosis, a careful investigation is needed to detect PTC when a fibroproliferative process is found in the thyroid gland. Furthermore, differential diagnosis of this variant from extensive fibrosis of other malignancies of the thyroid glands is important given that they also show an aggressive clinical course similar to a diffuse sclerosing variant of PTC and carcinosarcoma. The stroma in the diffuse sclerosing variant of PTC exhibits abundant lymphocytic infiltrate and psammoma bodies but a little fibroblastic proliferation. In PTC-FLS, stromal fibrosis is not a prognostic factor, and its behavior is similar to the other forms of PTC of the thyroid gland. In the CT imaging, thyroid fibromatosis shows a rich blood supply, but this finding cannot differentiate thyroid fibromatosis from thyroid cancer. PTCFLS can be definitively diagnosed by an intra-operative frozen examination and post-operative paraffin sections. As a low-grade malignant tumor, PTCFLS needs surgical management (7).

However, distant metastasis has not yet been reported for PTCFLS (8-10). Complete excision is needed. The recurrence rate of 2045% for local excision has been reported. Local excision along with radiation therapy can be used for efficient management of patients who are not willing to undergo surgery. Although previous studies have demonstrated the ineffectiveness of radiotherapy, radiation therapy is preferred (6). Non-surgical treatments include using systemic antiestrogens, non-steroidal anti-inflammatory drugs (NSAIDs), interferons, and other medications such as methotrexate along with doxorubicin. According to a previous study, a lowdosage of methotrexate combined with vinblastine in chemotherapy could lead to a 70% relapse-free survival rate over ten years (11). The prognosis of this malignancy depends on the size and location of the tumor. The 10-year survival rate of fibromatosis is typically 94%, and its 20-year survival rate is 86%.

Nevertheless, its survival rate is lower in patients suffering from aggressive fibromatosis affecting vital organs or large blood vessels (1).

In this study, we presented a 28-year-old female who was referred to the ENT clinic for a painless mass on the anterior part of the neck. The size of mass had shown gradually increase over the last 6 months. The thyroid function tests were normal. Ultrasound findings showed an 84×36 mm heterogeneous nodule in the right thyroid lobe without calcifications but increased vascularity. There were also some reactive lymph nodes in both sub-mandibular areas. Ultrasound-guided fine-needle aspiration of the right thyroid lobe nodule revealed a benign thyroid adenomatoid nodule. The patient underwent a right thyroid lobectomy. Intra-operative frozen section examination deferred to permanent sections and final pathologic studies confirmed a diagnosis of PTC with exuberant fibromatosis-like stroma. After 17 days, the patient was asymptomatic, and re-evaluation of the left thyroid lobe and follow-up were recommended.

## Conclusion

In this study, a rare variant of PTC, i.e., PTC-FLS, was diagnosed in a patient through combination of ultrasonography, fine-needle aspiration cytology, and histological examination.

## Conflict of Interest

Authors declared no conflict of interest.

## Funding

None.
